# Severe multiorgan toxicities after the first cycle of standard-dose etoposide–cisplatin therapy for metastatic nonseminoma successfully managed with etoposide–carboplatin: a case report

**DOI:** 10.1186/s13256-026-05894-3

**Published:** 2026-03-04

**Authors:** Takanori Mochizuki, Norifumi Sawada, Yoshihiro Nakanishi, Satoru Kira, Takahiko Mitsui

**Affiliations:** Department of Urology, Interdisciplinary Graduate School of Medicine, University of Yamanashi, Chuo-Shi, Shimokato 1110, Chuo, Yamanashi 409-3898 Japan

**Keywords:** Testicular nonseminoma, Etoposide–cisplatin, Cisplatin toxicity, Carboplatin, Case report

## Abstract

**Background:**

The combination of bleomycin, etoposide, and cisplatin is the standard regimen for good-risk metastatic nonseminomatous germ cell tumors, with etoposide and cisplatin used when pulmonary risk is present. Etoposide and cisplatin therapy is generally well tolerated, and severe toxicities are particularly rare after the first standard-dose cycle. We report a case of early-onset, simultaneous multiorgan failure after a single cycle of etoposide and cisplatin therapy, successfully managed with etoposide–carboplatin.

**Case presentation:**

A 54-year-old Japanese man with stage IIa nonseminomatous germ cell tumor received a standard-dose first cycle of etoposide and cisplatin therapy (etoposide 100 mg/m^2^, cisplatin 20 mg/m^2^, days 1–5). Despite appropriate hydration, he developed hepatic dysfunction, severe diarrhea, ototoxicity, acute kidney injury requiring hemodialysis, pancytopenia, and visual impairment. Supportive therapy, including dialysis, corticosteroids, broad-spectrum antibiotics, and granulocyte colony-stimulating factor, was provided. Although renal function partially improved, the patient had persistent severe renal dysfunction consistent with Kidney Disease: Improving Global Outcomes stage 3 acute kidney injury, rendering further cisplatin therapy unsafe. He subsequently received three cycles of etoposide–carboplatin (etoposide 100 mg/m^2^, days 1–5; carboplatin area under the curve 5), which were well tolerated. Tumor markers normalized, and imaging confirmed complete remission. At 12-month follow-up, he remained disease-free with stable renal function and improved general condition.

**Conclusion:**

This case demonstrates that even a standard-dose, first-cycle etoposide and cisplatin regimen can trigger abrupt, life-threatening multiorgan toxicities affecting renal, hepatic, auditory, hematologic, and visual systems. Such early, simultaneous onset has rarely been described in scientific literature. When cisplatin continuation is unsafe, etoposide–carboplatin may serve as a feasible alternative to achieve remission while minimizing systemic toxicity. Careful evaluation of patient-specific risk factors, including hypertension, renal reserve, and renin–angiotensin system inhibitor use, is essential before initiating cisplatin-based therapy. This case highlights the importance of individualized treatment planning and prompt recognition of severe chemotherapy-induced toxicities.

## Background

Testicular germ cell tumors (TGCTs) are the most common solid malignancies in young and middle-aged men, with cure rates exceeding 90% when treated appropriately [1]. For good-risk stage IIa nonseminomatous TGCTs (NSGCTs), three cycles of bleomycin, etoposide, and cisplatin (BEP) remain the standard regimen, whereas four cycles of etoposide and cisplatin (EP) are recommended for patients in whom bleomycin is contraindicated [2, 3]. Both regimens have been validated in large clinical studies and are considered highly effective with generally manageable toxicities.

Cisplatin is a key component of these regimens and is known to cause nephrotoxicity [4, 5], ototoxicity [6], hepatotoxicity, and myelosuppression [7], typically in a dose-dependent manner over multiple treatment cycles. However, the abrupt and simultaneous development of multiorgan toxicities—including renal, hepatic, auditory, hematologic, and visual dysfunction—during the first standard-dose EP cycle is exceedingly rare, and only isolated reports exist.

When cisplatin must be discontinued owing to severe toxicity, etoposide–carboplatin (E-Carbo) can be used as an alternative, although its efficacy is generally considered inferior to cisplatin in good-risk disease [8].

Here, we describe a rare and clinically significant case of good-risk NSGCT that developed severe multiorgan dysfunction shortly after completing the first EP cycle, ultimately requiring a switch to E-Carbo. This case highlights the unpredictable spectrum of early cisplatin-related toxicities and provides insight into practical management strategies for similar situations.

## Case presentation

**Fig. 1:**
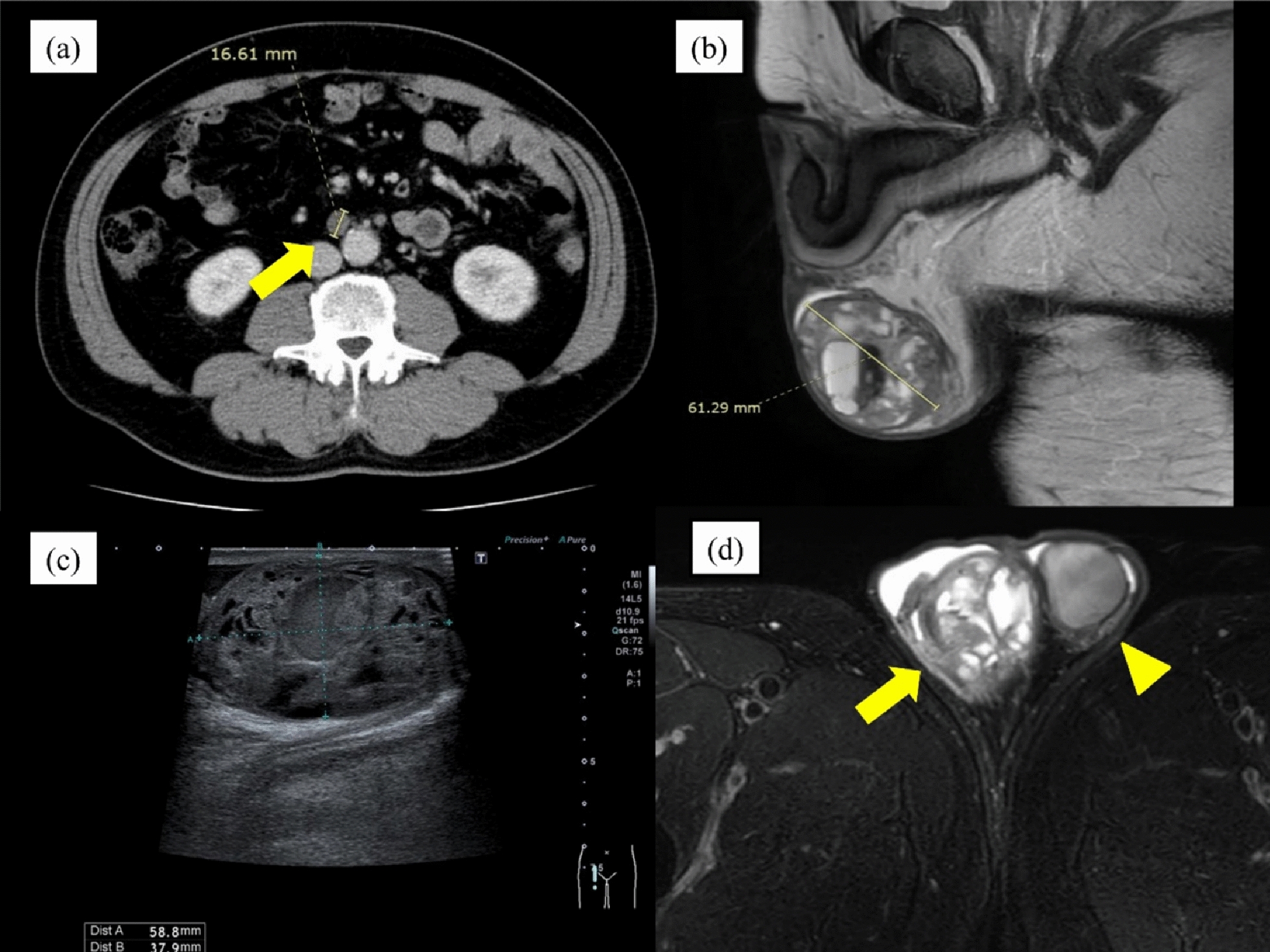
Imaging findings. **a** Contrast-enhanced computed tomography showing para-aortic lymph node metastasis (arrow). **b** Magnetic resonance imaging T2-weighted sagittal image. **c** Right testicular ultrasonography. **d** Magnetic resonance imaging T2-weighted coronal image; arrow indicates right testis, arrowhead indicates left testis

**Fig. 2:**
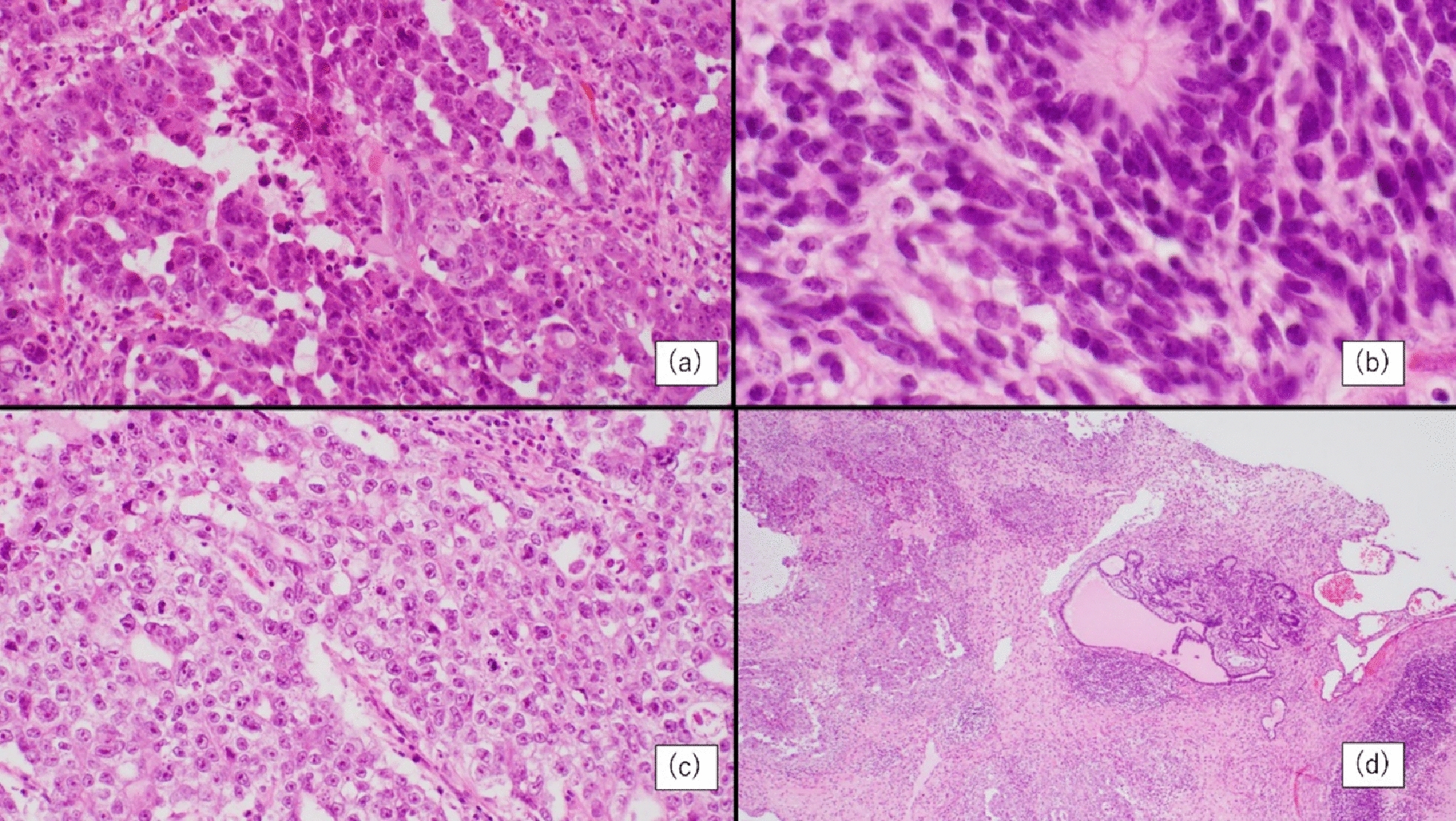
Histopathology of the left testis. **a** Teratoma (200×). **b** Embryonal carcinoma (200×). **c** Yolk sac tumor (200×). **d** Low-power view of the left testis (40×)

A 54-year-old Japanese man with hypertension and a 30-pack-year smoking history presented with progressive bilateral scrotal swelling for 2 months. He had no family history of malignancy and was taking a fixed-dose angiotensin receptor blocker (ARB)/calcium-channel blocker combination. This medication was discontinued at transfer to our hospital and was not resumed during subsequent chemotherapy. The physical examination was notable only for bilateral scrotal enlargement. Baseline tumor markers were as follows: lactate dehydrogenase (LDH) 325 U/L (reference: 124–222), alpha-fetoprotein (AFP) 668 ng/mL (reference: less than 10), and human chorionic gonadotropin (hCG) 1960 mIU/mL (reference: less than 2). Scrotal ultrasonography demonstrated heterogeneous enlargement of both testes, and contrast-enhanced computed tomography (CT) revealed bilateral testicular tumors with para-aortic lymphadenopathy up to 2.0 cm (Fig. 1). The patient subsequently underwent bilateral radical orchiectomy. Histopathology of the left testis showed the mixed nonseminomatous germ cell tumor consisting of embryonal carcinoma (50%), yolk sac tumor (40%), teratoma (10%), and focal trophoblastic elements (Fig. 2). The right testis showed chronic orchitis. On the basis of the imaging and pathology, the disease was classified as stage IIa (pT2N1M0S1), good-risk per the International Germ Cell Consensus Classification (IGCCC) criteria. Because of his smoking history, bleomycin was avoided, and EP therapy was initiated.

Etoposide–cisplatin (EP) chemotherapy was initiated on postoperative day (POD) 33 (hereafter referred to as EP day 1). During the first EP cycle, multiple toxicities developed. By EP day 4 (POD 36), hepatotoxicity appeared, followed by watery diarrhea and tinnitus on EP day 5 (POD 37). On the sixth day, hearing impairment developed. Upon consultation with an otolaryngology department specialist, a diagnosis of bilateral high-frequency sensorineural hearing loss (right 55 dB, left 50 dB) was made. Treatment with steroid was then initiated. By EP day 7 (POD 39), the patient developed anuric acute kidney injury requiring hemodialysis. The patient had not previously received heparin; however, unfractionated heparin was used as the anticoagulant during hemodialysis at the referring hospital from EP day 7 to EP day 9.

On EP day 9 (POD41), pancytopenia with febrile neutropenia developed (hemoglobin 7.6 g/dL, platelets 12 × 10^9^/L, white blood cell (WBC) 0.9 × 10^9^/L). By EP day 10 (POD42), the platelet count nadired at 9 × 10^9^/L. On EP day 14 (POD 46), a platelet factor 4 (PF4) antibody assay (IgG/A/M; optical density 1.0) returned positive. Heparin was therefore discontinued, and all subsequent dialysis sessions at our hospital used argatroban. His platelet count improved to 92 × 10^9^/L by EP day 18 (POD 50), consistent with heparin-induced thrombocytopenia (HIT) based on serologic results and clinical recovery.

The patient also reported blurred vision and photophobia. On EP day 10 (POD 42), he was transferred to our hospital. At transfer, laboratory tests showed severe renal impairment (blood urea nitrogen (BUN) 119.4 mg/dL, creatinine 11.88 mg/dL), hepatic injury (ALT 166 U/L, γ-GTP 559 U/L), and pancytopenia (WBC 700/μL, Hb 8.8g/dL, platelets 16,000/μL). Although the patient demonstrated marked elevations in liver enzymes, he did not exhibit clinical signs of hepatic dysfunction, such as jaundice, coagulopathy, or altered mental status. We therefore interpreted this finding as drug-induced liver injury (DILI) without overt hepatic dysfunction.

On EP day 9, the patient reported bilateral photophobia and subjective visual field constriction. He was evaluated by an ophthalmologist on EP day 10. Best-corrected visual acuity was 0.3 in the right eye and 0.6 (pinhole, no correction) in the left eye. Intraocular pressure measured 13.0 mmHg in the right eye and 15.0 mmHg in the left eye. Funduscopic examination revealed bilateral macular edema without evidence of retinal hemorrhage or optic disc swelling.

To exclude central nervous system involvement, brain magnetic resonance imaging performed on EP day 15 demonstrated no findings suggestive of optic neuritis or intracranial pathology. At follow-up on EP day 21, repeat ophthalmologic examination showed a marked improvement of the macular edema, accompanied by the resolution of photophobia and visual field constriction. At a subsequent visit on EP day 57, best-corrected visual acuity had further improved to 0.9 in both eyes.

A thorough infectious workup was performed because of febrile neutropenia. Blood cultures obtained on EP day 10 remained negative, and chest radiography showed no pneumonia. Although the patient was oliguric, urinalysis from limited urine output showed no bacteria or pyuria, making urinary tract infection unlikely; urine culture could not be obtained. Empirical antibiotics were initiated—vancomycin on EP day 14 (POD 46) and piperacillin–tazobactam on EP day 15 (POD 47)—in accordance with febrile neutropenia guidelines (Japanese Society of Medical Oncology (JSMO) guidelines). Repeat blood cultures grew *Staphylococcus epidermidis* in one of two bottles, interpreted as contamination rather than true bacteremia.

Supportive management included renal replacement therapy, hematologic support, and anti-inflammatory treatment. Granulocyte colony-stimulating factor was administered as filgrastim at a dose of 75 μg/day subcutaneously from EP day 10 to day 14, followed by an increased dose of 150 μg/day on EP days 15 and 16 in response to persistent neutropenia. Systemic corticosteroid therapy was initiated on EP day 6 with oral betamethasone (Lindelon^®^) at 12 mg/day for suspected inflammatory involvement of the auditory and ocular systems, tapered to 8 mg/day on EP day 8, and discontinued on EP day 17 as clinical and laboratory parameters improved.

Renal replacement therapy was initiated on EP day 7 because of severe oliguric acute kidney injury and uremia. Intermittent hemodialysis was performed on five consecutive days, followed by three additional sessions on an every-other-day schedule, after which dialysis was successfully discontinued as urine output and metabolic parameters recovered. However, severe renal dysfunction consistent with KDIGO stage 3 acute kidney injury persisted.

Given the severity of cisplatin-associated toxicities, cisplatin was permanently discontinued. A cisplatin-free regimen, etoposide–carboplatin (E-Carbo), was initiated. Carboplatin was dosed using Calvert’s formula (area under the curve (AUC) 5, based on an eGFR of 19 mL/min/1.73 m^2^ at recovery), and etoposide 100 mg/m^2^ was administered on days 1–5. Three cycles were completed without significant complications, with only mild myelosuppression observed.

**Fig. 3:**
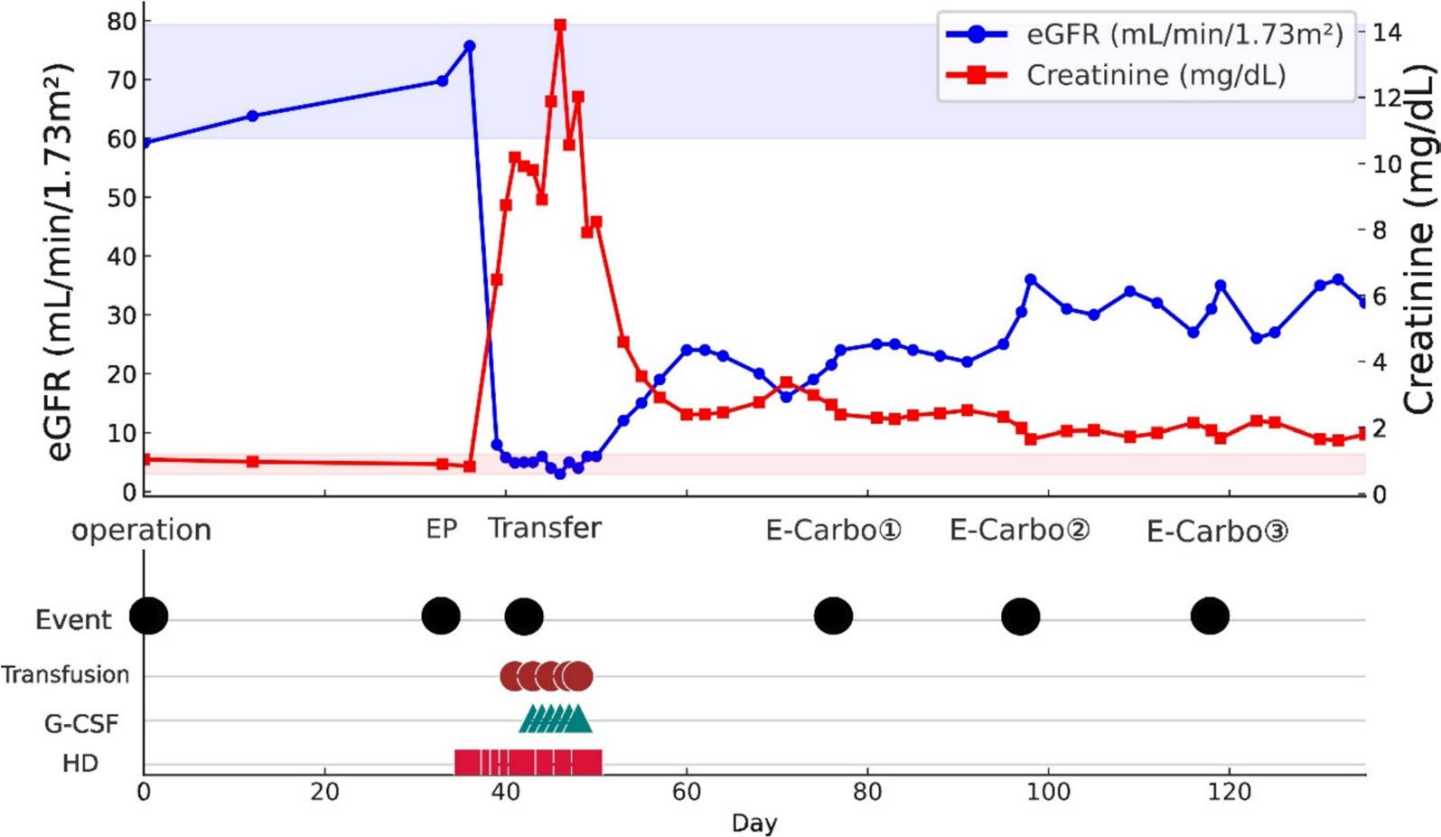
Clinical course and timing of adverse events during etoposide–cisplatin and subsequent etoposide–carboplatin therapy. Day 0 indicates the day of bilateral orchiectomy. Etoposide–cisplatin day 1 corresponds to postoperative day 33. The timeline is presented using both postoperative day (counted from surgery) and etoposide–cisplatin day (counted from initiation of etoposide–cisplatin therapy) to clarify the temporal relationship between surgery, chemotherapy, and adverse events

Tumor markers normalized (LDH 208 U/L; AFP 3.7 ng/mL; hCG 0.2 mIU/mL), and positron emission tomography (PET)-CT demonstrated complete metabolic remission. At 12-month follow-up, the patient remained disease-free, with stable renal function (chronic kidney disease (CKD) stage IV) and Eastern Cooperative Oncology Group (ECOG) performance status of 1. Figure 3 depicts the overall clinical timeline. Table 1 outlines the day-by-day emergence of complications, and Table 2 summarizes the onset, management, and outcomes of each adverse event.

**Table 1:** Timeline of major adverse events and treatment course

Day/week	Event	Findings/actions
Days 1–5	EP first cycle (etoposide 100 mg/m^2^ + CDDP 20 mg/m^2^)	Standard hydration
Days 4–7	Diarrhea, hearing loss, AKI,	Multiple organ failure/otolaryngologist and nephrologist consultation
Day 7	Liver damage, AKI	Hemodialysis initiated
Day 9	Pancytopenia, febrile neutropenia, HIT^+^	Granulocyte colony stimulating factor (G-CSF), antibiotics, transfusions blood and platelet
Day10	Photophobia	Ophthalmology consult
Week 3	Dialysis discontinued	CKD stage IV remained
After recovery	E-Carbo × 3 cycles (AUC 5)	Tolerated, markers normalized
12 months	Disease-free	Stable

**Table 2:** Summary of complications observed during and after EP therapy

Complications	Grade	Duration of illness (days)	Treatment	Outcome
Febrile neutropenia	3	10–16	G-CSF, antibiotics	Improved
Anemia	2	17–21	Transfusion, erythropoietin	Improved
Platelet count decreased (HIT antibody positive)	4	9	Platelet transfusion	Improved
Hearing impairment	3	6	Steroid	Improved, but symptoms remain
Visual impairment	3	9	Follow-up	Improved, but symptoms remain
Photophobia	3	9	Follow-up	Improved, but symptoms remain
Hepatobiliary disorder	4	4	Follow-up	Improved
Peripheral sensory neuropathy	2	7	Vitamin B12	Improved, but symptoms remain
Diarrhea	2	5–13	Follow-up	Improved
Acute kidney injury	4	11	Hemodialysis	Weaned off hemodialysis, but CKD stage 4 renal impairment remains

Adverse events were listed according to the Common Terminology Criteria for Adverse Events (CTCAE) version 5.0, including duration, treatment interventions, and outcomes

## Discussion

The present case describes a patient with good-risk stage IIa nonseminomatous germ cell tumor (NSGCT) who developed unusually severe and simultaneous multiorgan toxicities after the first cycle of etoposide–cisplatin (EP) therapy [9]. Although cisplatin is well known for its nephrotoxicity [10], ototoxicity [6], and myelosuppressive effects [7], the abrupt onset of concurrent renal failure, hepatotoxicity, pancytopenia, auditory impairment, gastrointestinal toxicity, and visual disturbance [11] within a single treatment cycle is exceedingly rare. Only isolated case reports in existing literature describe such early and broad toxicity clusters, particularly in patients with otherwise good-risk NSGCT receiving guideline-concordant therapy [12]. This case underscores the unpredictable and potentially life-threatening nature of cisplatin-associated adverse events, even when administered at standard doses and in the absence of classical high-risk features.

EP remains an established first-line regimen for good-risk NSGCTs, especially in individuals for whom bleomycin is relatively contraindicated, such as those with a smoking history or preexisting pulmonary conditions [13]. Large phase III trials and observational studies have demonstrated comparable efficacy between BEP and four cycles of EP, with EP often regarded as the safer alternative. Cisplatin, however, remains one of the most toxic cytotoxic agents in contemporary oncology practice. Its toxicities are typically dose-dependent and cumulative, yet in rare cases, severe toxicity may present precipitously after the first infusion. The breadth of toxicities observed in this patient highlights the need for clinicians to remain vigilant for atypical and early manifestations of cisplatin injury.

Cisplatin exerts its antineoplastic effect primarily through the formation of DNA intra- and interstrand crosslinks, leading to apoptosis in rapidly dividing tumor cells; however, this same mechanism also underlies toxicity in normal tissues [9, 14, 15]. Nephrotoxicity arises predominantly from direct tubular epithelial injury mediated by oxidative stress, mitochondrial dysfunction, impaired DNA repair pathways, and activation of apoptotic signaling [16, 17]. Beyond direct epithelial toxicity, increasing experimental and clinical evidence indicates that cisplatin induces vascular endothelial injury, characterized by endothelial cell apoptosis, nitric oxide depletion, microvascular dysfunction, and increased vascular permeability, which may impair tissue perfusion and promote ischemic and inflammatory damage across multiple organ systems [18–20]. This endothelial-centered mechanism provides a unifying explanation for the simultaneous multiorgan involvement observed in our patient. Interindividual susceptibility to cisplatin toxicity is increasingly recognized to be influenced by genetic factors affecting drug transport, detoxification, and DNA repair [21]. Variants in solute carrier family 22 member 2 (*SLC22A2/OCT2*), which regulates renal uptake of cisplatin, and in excision repair cross-complementing genes (*ERCC1/ERCC2*), involved in nucleotide excision repair, have been reproducibly associated with increased risks of nephrotoxicity and ototoxicity [22, 23]. In addition, the multidrug and toxin extrusion transporter 1 (MATE1), encoded by solute carrier family 47 member 1 (*SLC47A1*), mediates apical efflux of cisplatin and its metabolites in renal tubular cells, and genetic variation in this pathway has been linked to altered cisplatin handling and heightened renal vulnerability [21]. Detoxification pathways mediated by glutathione *S*-transferase isoenzymes, including *GSTP1* and *GSTM1*, further modulate susceptibility through conjugation of reactive platinum species, with low-activity or null variants associated with increased systemic toxicity [21, 23]. Although pharmacogenomic testing was not performed in this case, the unusually severe and multisystem toxicities observed raise the possibility that an underlying genetic predisposition, in combination with cisplatin-induced endothelial dysfunction, contributed to the patient’s clinical course [24].

To better define the rarity and educational value of early multiorgan toxicity after cisplatin-based chemotherapy, we conducted a targeted literature search for reports published in the last 20 years describing standard-dose cisplatin (≤ 100 mg/m^2^ per cycle) associated with grade ≥ 3 toxicity affecting two or more organ systems within the first or second cycle. Although several reports of cisplatin nephrotoxicity, ototoxicity, or neurotoxicity exist, very few described simultaneous involvement of renal, hepatic, hematologic, auditory, and ocular systems.

This lack of comparable cases underlines the uniqueness of our report and supports its contribution to increasing clinician awareness of potentially unpredictable, severe cisplatin toxicity—even in good-risk, guideline-concordant settings. A summary table of comparable published cases is presented (Table 3) [25–31], highlighting differences in patient background, regimen, affected organs, and outcomes.

**Table 3:** Summary of cisplatin induced toxicity case report

References	Primary malignancy	Regimen/cisplatin dose	Cycle at toxicity onset	Organs affected (grade ≥ 3)	Reported risk factors	Outcome
Martin [37]	Epidermoid carcinoma	Cisplatin (100 mg/m^2^, day 1) and 5-fluorouracil (1 g/m^2^/day intravenous days 2–6)	–	Heart	None	Died
Sekine [26]	Non-small-cell lung cancer	Cisplatin 80 mg/m^2^ (122 mg/ body) on day 1, vinorelbine 20 mg/m^2^ (32 mg/body) on days 1 and 8, and thoracic radiotherapy 30 Gy/15 fraction	Day ~36 after first cisplatin administration	Meningitis	None	Saved
Cherniawsky [28]	Cholangiocarcinoma	Cisplatin 34 mg (20 mg/m^2^), and gemcitabine was dosed at 1700 mg (1000 mg/m^2^)	Day ~150 after chemotherapy initiation	Encephalopathy syndrome	Gemcitabine	Saved
Aaron [29]	Urothelial carcinoma	Cisplatin and gemcitabine (no dosage information provided)	Day 2 after first cisplatin administration	Syndrome of inappropriate antidiuretic hormone secretion (SIADH)	Ibuprofen, pulmonary and cerebral disease, prior nephroureterectomy	Saved
Matsumoto [30]	Lung cancer (squamous cell carcinoma)	Cisplatin/S-1	Day 13 after first cisplatin administration	Intramural thrombus	None	Saved
Josu [31]	Germ cell tumors	Cisplatin + etoposide	~30 days after chemotherapy initiation	ST-elevation acute coronary syndrome, pulmonary thromboembolism with pulmonary infarction	Dyslipidaemia, arterial hypertension, and obesity	Saved
Mochizuki 2025 (our case)	Germ cell tumors	Cisplatin (20 mg /m^2^) + etopside (100 mg/m^2^)	Day 4 after first cisplatin administration	Liver injury, acute kidney disease, visual disturbance, auditory disturbance, neutropenia, low platelet	Angiotensin receptor blocker (ARB), hypertension, chronic smoking	Saved

References are numbered according to the reference list in the main manuscript. The last row corresponds to the present case

In the present case, the patient’s history of hypertension, chronic smoking, and long-term use of a renin–angiotensin system inhibitor (angiotensin II receptor blocker) may have rendered his microvasculature particularly vulnerable (“perfect storm”). The added insult of cisplatin-induced endothelial dysfunction could therefore have tipped homeostasis into widespread organ injury. This hypothesis may explain why a “standard-dose” EP cycle—normally considered safe—resulted in severe multiorgan toxicity in this patient.

Hepatotoxicity, although less common, has been documented and may relate to oxidative stress within hepatocytes, mitochondrial damage, and microvascular inflammatory changes [32]. Ototoxicity, typically resulting from injury to cochlear outer hair cells and the stria vascularis, is often irreversible [33, 34]. Bone marrow suppression is a well-recognized adverse effect, reflecting the susceptibility of rapidly dividing hematopoietic progenitor cells to DNA-damaging agents [35].

While each toxicity individually is well described in literature, their simultaneous occurrence after a single cycle—as seen here—is extraordinary. Visual toxicity from cisplatin is extremely uncommon, and drug-induced macular edema is considered one of the rarest manifestations. Only a handful of case reports describe cisplatin-associated retinal injury, including optic neuropathy, macular edema, or neurosensory retinal detachment. Cisplatin-related visual disturbances were attributed to oxidative stress–mediated retinal injury, ischemic dysfunction of the retinal microvasculature, and toxic effects on the retinal pigment epithelium (RPE) [36, 37]. These mechanisms are consistent with cisplatin’s recognized propensity to induce endothelial dysfunction and microvascular compromise in other organs.

In our patient, the diagnosis of drug-induced macular edema was confirmed by ophthalmologic evaluation, with no evidence of optic neuritis or infectious etiology.

Given the simultaneous renal, hepatic, and auditory toxicities, the presence of macular edema reinforces the notion that cisplatin-induced systemic endothelial injury played a central role in the multiorgan dysfunction. Moreover, the early onset of visual symptoms—occurring within the first treatment cycle—has been rarely documented and further underscores the unique severity of toxicity in this case.

An alternative and important diagnostic consideration in patients presenting with multiorgan dysfunction during chemotherapy is severe infection or sepsis, particularly in the setting of febrile neutropenia. In this case, sepsis was thoroughly evaluated as a potential primary or contributory cause. Blood cultures obtained promptly after fever onset were negative, chest radiography showed no pulmonary infection, and urinalysis revealed no pyuria or bacteriuria despite near-anuria. A repeat blood culture grew *Staphylococcus epidermidis* in only one of two bottles and was interpreted as likely contamination rather than true bloodstream infection. Although broad-spectrum antimicrobial therapy was appropriately administered, the temporal sequence of organ injury—beginning with hepatic dysfunction and auditory symptoms before neutropenia—was more consistent with early cisplatin toxicity than with sepsis-driven multiorgan failure. For these reasons, infection was deemed unlikely to be the unifying etiology of the patient’s condition.

Heparin-induced thrombocytopenia (HIT) represented another relevant differential diagnosis for the patient’s profound thrombocytopenia. The patient had been exposed to unfractionated heparin during hemodialysis at the referring hospital, and his subsequent sharp platelet decline, together with a positive platelet factor 4 antibody assay, was consistent with HIT [38]. Platelet recovery following heparin discontinuation further supported this diagnosis. Nonetheless, HIT alone could not account for the simultaneous renal, hepatic, auditory, gastrointestinal, and visual toxicities and therefore was considered contributory but not causative. These overlapping processes highlight the importance of evaluating both chemotherapy-induced toxicities and treatment-related complications when managing complex patient presentations.

Several baseline and treatment-related factors may have predisposed this patient to developing unusually severe cisplatin toxicity. Established risk factors for cisplatin-induced nephrotoxicity include older age, smoking history, reduced renal reserve, and exposure to medications that affect renal perfusion [39]. Although renin–angiotensin system inhibitors (RASi), such as angiotensin II receptor blockers (ARBs), are generally regarded as vasoprotective agents with well-established antihypertensive and anti-inflammatory effects, their influence on susceptibility to nephrotoxic injury may be context-dependent. In our case, the ARB was discontinued at the time of chemotherapy initiation, and therefore, ongoing RAS inhibition was unlikely to have directly contributed to the observed toxicities. We therefore hypothesize that the patient’s long-standing hypertension and chronic smoking contributed to a subclinical microvascular vulnerability, and that the superimposition of cisplatin-induced endothelial injury on this preexisting vascular milieu—rather than ARB exposure per se—may have synergistically amplified tissue damage and contributed to the unusually rapid and widespread toxicity observed [40].

When cisplatin must be discontinued owing to severe adverse events, treatment alternatives become essential. While cisplatin-based regimens such as etoposide, ifosfamide, and cisplatin (VIP) and vinblastine, ifosfamide, and cisplatin (VeIP) represent standard curative options for NSGCTs [13], their reliance on cisplatin renders them unsuitable for patients who develop severe cisplatin-induced toxicities. Carboplatin-based regimens have historically demonstrated inferior progression-free survival compared with cisplatin in good-risk NSGCTs; however, in specific clinical scenarios—particularly those involving life-threatening toxicity—carboplatin may offer an acceptable balance of safety and efficacy. In this case, the patient’s persistent renal dysfunction, auditory impairment, and residual visual disturbance made cisplatin re-challenge unsafe, and thus, etoposide–carboplatin (E-Carbo) was selected [8]. The regimen was well tolerated, and three cycles produced complete biochemical and radiologic remission. This outcome demonstrates that carboplatin-based therapy can preserve curative potential when cisplatin toxicity precludes further cisplatin exposure.

In summary, this case highlights several critical clinical principles. Cisplatin-induced toxicities can be sudden, severe, and multisystemic, even after a single treatment cycle in patients with good-risk nonseminomatous germ cell tumors. Preexisting microvascular vulnerability related to factors such as long-standing hypertension and chronic smoking may predispose patients to early and exaggerated endothelial and organ injury. Although cisplatin-based regimens remain the cornerstone of curative therapy, continuation of cisplatin may become unsafe in selected patients who develop life-threatening adverse events. In such scenarios, a carboplatin-based regimen can represent a pragmatic and patient-centered alternative that preserves curative intent while minimizing the risk of further organ damage. Ultimately, the timely recognition of evolving toxicity patterns, careful differential diagnosis, and rapid therapeutic modification are essential to optimize both patient safety and oncologic outcomes.

## Conclusion

This case highlights that severe, multiorgan cisplatin toxicity can occur even after a single standard-dose cycle in patients with good-risk NSGCT. Early recognition and timely transition to a carboplatin-based regimen may preserve curative intent while minimizing the risk of irreversible organ damage.

## Data Availability

All data supporting the findings of this study are included within the article.

## Abbreviations

AKI: Acute kidney injury
ARB: Angiotensin receptor blocker
BEP: Bleomycin, etoposide, and cisplatin
CKD: Chronic kidney disease
CT: Computed tomography
DILI: Drug-induced liver injury
DNA: Deoxyribonucleic acid
E-Carbo: Etoposide and carboplatin
EP: Etoposide and cisplatin
G-CSF: Granulocyte colony stimulating factor
eGFR: Estimated glomerular filtration rate
GSTP1: Glutathione *S*-transferase pi 1
GSTM1: Glutathione *S*-transferase mu 1
HIT: Heparin-induced thrombocytopenia
IGCCC: International Germ Cell Consensus Classification
JSMO: Japanese Society of Medical Oncology
KDIGO: Kidney Disease: Improving Global Outcomes
MATE1: Multidrug and toxin extrusion 1
NSGCTs: Nonseminomatous germ cell tumors
OCT2: Organic cation transporter 2
POD: Postoperative day
SLC22A2: Solute carrier family 22 member 2
SLC47A1: Solute carrier family 47 member 1
SNPs: Single nucleotide polymorphisms
RPE: Retinal pigment epithelium
WBC: White blood cell
